# Rheology and Amylase Resistance of Calcium-Enriched Calorie-Dense Emulsions for Dysphagia Management

**DOI:** 10.3390/gels12030192

**Published:** 2026-02-25

**Authors:** Huaiwen Yang, Yi-Zhen Shen

**Affiliations:** Department of Food Science, National Chiayi University, 300, Xuefu Rd., East Dist., Chiayi City 600355, Taiwan; s1110442@mail.ncyu.edu.tw

**Keywords:** dysphagia, locust bean gum, pre-gelatinized rice flour, α-amylase, apparent viscosity, texture property analysis

## Abstract

This study examines how changes in salivary α-amylase and calcium affect the rheological properties and stability of food emulsions thickened with Locust Bean Gum (LBG) and Pre-gelatinized Rice Flour (RF), containing 10% Medium-chain Triglycerides (MCT). Increasing the LBG: RF ratio improves resistance to simulated saliva dilution. The 1:4 (LBG: RF) emulsion maintained an apparent viscosity within the NDD nectar-like range after simulated saliva addition, making it suitable for patients with dysphagia. Calcium sources from lactate (LC) and gluconate (GC) enhanced the emulsion’s resistance to α-amylase degradation, especially at a shear rate of 10 s^−1^. The study also evaluated levels according to the International Dysphagia Diet Standardisation Initiative (IDDSI). Both NDD honey-like and pudding-like samples were classified as IDDSI Level 3 by the syringe test, showing that different grading systems and food compositions can lead to variations. After simulated saliva treatment, the 1:4 (LBG: RF) sample showed the least degradation. These findings highlight the significant role of salivary α-amylase in thickened foods and emphasize the importance of optimizing thickener types and formulation ratios to enhance dietary safety for individuals with dysphagia.

## 1. Introduction

Dysphagia, or difficulty swallowing, is increasingly recognized as a major healthcare concern worldwide, particularly as the population ages [1]. It affects about 8% of the global population, especially 10–30% of adults aged 65 and older, 30% of stroke survivors, 60–80% of patients with neurodegenerative conditions, and over 50% of elderly individuals in care facilities [2]. Dysphagia increases the risk of pulmonary aspiration, which can lead to aspiration pneumonia. The primary dietary management involves texture-modified foods or thickened liquids, especially for those with chronic dysphagia, as these provide additional time for airway protection during swallowing [3]. Thickeners are essential for controlling food texture effectively, managing water to provide structure, flow, stability, and mouthfeel [4]. Commercial market options include starch- or gum-based thickeners for treating dysphagia patients [5]. Commercial market options include starch- or gum-based thickeners for treating dysphagia patients. During oral processing, α-amylase primarily hydrolyzes starch. When food interacts with saliva containing α-amylase, viscosity can decrease significantly, particularly in starch-thickened foods, which increases the risk of aspiration in dysphagia patients. Starch-based thickeners generally require a higher amount than gum-based thickeners to achieve similar viscosity. While gum-based thickeners like Locust bean gum (LBG) produce highly viscous aqueous solutions at relatively low concentrations and are stable across a wide pH range, they do not inherently address nutritional deficiencies, such as low calcium intake, which are common among the elderly. To address these nutritional hurdles, calcium fortification using organic acid salts, such as calcium lactate (LC) and calcium gluconate (GC), offers a promising approach. Unlike inorganic sources like calcium carbonate, which may remain as insoluble particles in thickened food systems, LC and GC are characterized by higher solubility and intestinal bioavailability within complex food matrices [6]. Furthermore, the choice of calcium sources is critical as it may influence the structural integrity of the thickener; for instance, research has shown that organic calcium salts can alter starch conformation and affect the overall rheological behavior of modified rice systems [7]. Additionally, since calcium ions at body temperature may modulate the activity of salivary α-amylase, the selection of highly soluble organic forms is essential to balance nutritional delivery with the maintenance of a stable bolus viscosity during oral processing [8]. Therefore, there is a need for advanced formulations, such as the LBG-RF-MCT emulsion, to overcome the limitations of simple starch degradation while providing essential nutrients.

During oral processing, α-amylase primarily hydrolyzes starch. When food interacts with saliva containing α-amylase, viscosity can decrease significantly, particularly in starch-thickened foods, increasing aspiration risk in dysphagia patients [9]. Since saliva plays a critical role in safe swallowing, its effects on thickened foods are often understudied. Therefore, studying the resistance of dysphagia products to salivary hydrolysis is crucial for effective management [6,9,10].

Locust bean gum (LBG) has been classified as “Generally Recognized as Safe” (GRAS) by the U.S. Food and Drug Administration and is listed as E410 [11,12]. Composed mainly of mannose and galactose, LBG achieves optimal solubility upon heating. Due to its natural origin and stability across a wide pH range, it is widely used as a stabilizer and thickener in foods and is often included in dysphagia diets. LBG can produce highly viscous aqueous solutions at relatively low concentrations, enhancing texture and other functional properties by controlling the aqueous phase. Additionally, LBG can form strongly birefringent structures at the oil-water interface, stabilizing emulsions by creating a liquid-crystalline layer around droplets [13,14]. According to the “Nutrition and Health Survey in Taiwan (NAHSIT 2017–2020),” calcium intake remains the lowest among the Taiwanese population [15]. The elderly and postmenopausal women, who are at higher risk of calcium loss from bones, require special attention to their calcium intake. Besides naturally calcium-rich foods, calcium-fortified foods or supplements are additional options. Moreover, studies suggest that calcium added at 37 °C may enhance salivary amylase activity [8,16]. In high-risk elderly populations with dysphagia, reduced digestive function and restricted food textures hinder nutrient absorption and metabolism, leading to decreased food intake. This creates a vicious cycle involving malnutrition, sarcopenia, and aspiration pneumonia.

Therefore, developing nutritious foods that meet dysphagia standards is essential for promoting optimal nutrition. Lipids are calorie-dense and are often added to foods to increase energy content. Medium-chain triglycerides (MCTs), compared to long-chain fatty acids found in most dietary fats, are nearly tasteless and odorless, have less impact on blood lipids, and are absorbed and metabolized more quickly and efficiently. Additionally, MCT intake has been shown to increase the activation of acetylated growth hormone-releasing peptides (GHRPs), which enter the bloodstream and stimulate growth hormone (GH) release from the brain. GH can promote food intake and muscle growth, while GHRPs can also increase gastric motility, stimulate gastric acid secretion, and regulate insulin secretion [17,18]. Shear rates between 10 and 50 s^−1^ are suitable for simulating oral conditions, where perceived viscosity in sensory tests correlates most closely with apparent viscosity measured at 10 s^−1^. The National Dysphagia Diet (NDD) standards, published by the Academy of Nutrition and Dietetics in 2002, classify dysphagia products based on apparent viscosity at 50 s^−1^ [19,20,21]. The International Dysphagia Diet Standardisation Initiative (IDDSI) established an international standardized terminology for texture-modified foods and thickened liquids for patients with dysphagia [19]. This standard includes eight levels (0–7), with beverages classified from Level 0 to 4, and foods from Level 3 to 7 [22]. Notably, IDDSI grading is designed for frontline staff such as nurses, patients, or caregivers; hence, it relies on subjective assessments rather than objective measurements to determine liquid thickness.

However, unquantified values may make its grading standards unsuitable for food manufacturing or research. Relying only on qualitative methods cannot detect subtle differences within food or liquid textures at the same IDDSI level, and the lack of quantitative data limits researchers’ ability to study how food texture affects swallowing [22,23]. The objectives of this study are to: (1) determine the best LBG addition ratio and add MCT to develop a pre-absorptive food emulsion resistant to amylase; (2) assess how oral processing influences the apparent viscosity and texture of the easy-to-absorb food emulsion and those modified with different calcium sources; (3) classify the easy-to-absorb food emulsion according to the IDDSI framework and observe differences using a texture analyzer with IDDSI fork (A/IDDSI-F) and spoon (A/IDDSI-SC) probes to produce quantitative data for IDDSI grading as an objective standard; and (4) explore the rheological properties, microstructure, color, and storage stability of the easy-to-absorb food emulsion.

## 2. Results and Discussion

### 2.1. Rheological Properties of Food Emulsion

#### 2.1.1. Apparent Viscosity Trend

The finding that L1R0.5 requires the lowest thickener concentration to reach target viscosities suggests a higher thickening efficiency compared to L1R4 and L1R8. This can be attributed to the inherent properties of Locust Bean Gum (LBG), which is known to produce highly viscous solutions at low concentrations through molecular chain entanglement. In contrast, starch-based thickeners like Pre-gelatinized Rice Flour (RF) generally need a significantly higher concentration to achieve similar viscosity. In formulations with higher RF ratios (e.g., L1R8), the RF acts as a filler that requires an increase in total solid content to maintain the desired rheological properties, effectively diluting the high-efficiency hydrocolloid network provided by LBG. Higher shear viscosity is associated with a longer pharyngeal transit time, causing the liquid to flow more slowly through the oropharynx. This creates a longer window for tracheal closure. Thickeners interact with water molecules to form a three-dimensional hydrated network, increasing viscosity through molecular chain entanglement. This is vital for dysphagia patients, as it helps control swallowing muscles, quickly close the airway, and open the food passage, thereby reducing the risk of aspiration. The National Dysphagia Diet (NDD) framework [24] categorizes thickened products based on their apparent viscosity (*η_app_*) measured at a shear rate of 50 s^−1^.

**Figure 1:**
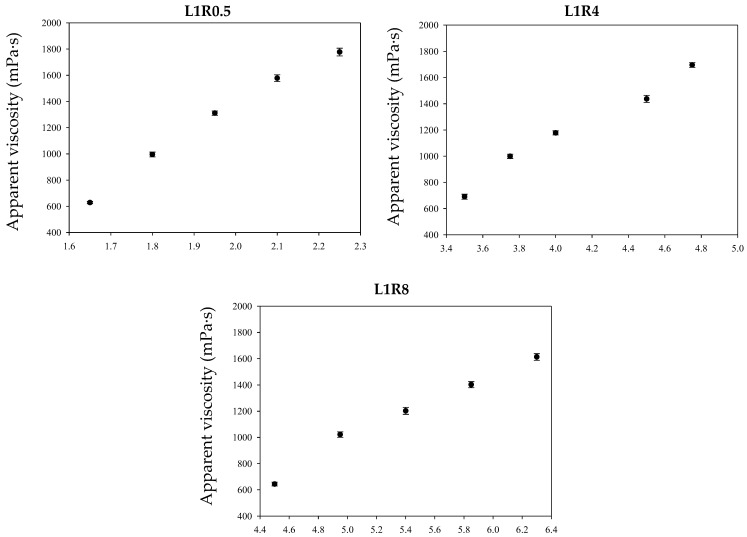
Concentration (*w*/*w*%) and apparent viscosity (mPa·s) of L1R0.5, L1R4, and L1R8 food emulsions. Formulas are shown in Section 4.2.2. Error bars represent standard deviations (n = 3).

Figure 1 illustrates the relationship between the concentration of LBG-RF food emulsions and their apparent viscosity at 50 s^−1^ for different blending ratios: L1R0.5, L1R4, and L1R8. When comparing food emulsions with different LBG-RF ratios, L1R0.5 required the lowest thickener concentration to reach the same apparent viscosity, followed by L1R4, while L1R8 required the highest. This indicates that L1R0.5 has the most efficient thickening capability. This finding aligns with Calmarza-Chueca et al. [25], who indicated that starch-based thickeners generally need a higher amount than gum-based thickeners to achieve similar viscosity.

Regression analysis using the Power law and Linear models was performed on food emulsion concentration versus apparent viscosity at 50 s^−1^. A consistent upward trend was observed across all food emulsions, indicating that apparent viscosity increased linearly with emulsion concentration. R^2^ values ranged from 0.988 to 0.994, as summarized in Table 1.

#### 2.1.2. Sample Formulation for Further Analysis

To ensure product feasibility and account for the detection limits of the instrumentation, this study adjusted the food emulsions to achieve target apparent viscosities of 1050 ± 50 mPa·s (honey-like consistency) and 1800 ± 50 mPa·s (pudding-like consistency) for subsequent experiments. Furthermore, the effects of different calcium sources—calcium carbonate (CC), calcium lactate (LC), and calcium gluconate (GC)—on the LBG-RF food emulsions are based on the HL1R0.5 formula listed in Section 4.2.2.

#### 2.1.3. Shear-Dependent Viscosity and Flow Behavior

**Figure A1:**
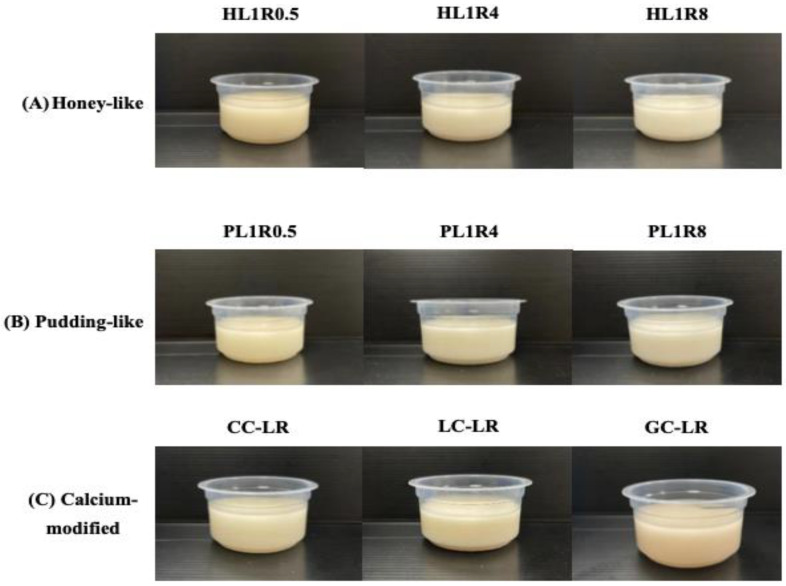
Visual properties of food emulsions: (**A**) Honey-like, (**B**) Pudding-like, (**C**) Calcium-modified. Formulas are shown in Table 6.

Previous research has shown that a shear rate of 10 s^−1^ is relevant to in vivo oral perception of viscosity [18]. The apparent viscosities of the prepared food emulsions at shear rates of 50 s^−1^ and 10 s^−1^ are shown in Table 2. At both shear rates, 50 s^−1^ and 10 s^−1^, the results indicate that adding CC and GC increased the apparent viscosity of the HL1R0.5 formulation. Conversely, the addition of LC decreased it. The study by Wariyah et al. [7] demonstrated that rice modified with LC had a lower apparent viscosity than regular rice, suggesting that calcium addition may alter starch conformation. Comparing the apparent viscosities of all food emulsions at 50 s^−1^ and 10 s^−1^ revealed that viscosity at 10 s^−1^ was consistently lower than at 50 s^−1^. This difference indicates that all food emulsions exhibit shear-thinning behavior. Visual images showing the rheological appearance of each food emulsion are included in Figure A1.

#### 2.1.4. Rheological Modeling and Flow Behavior Analysis

**Figure 2:**
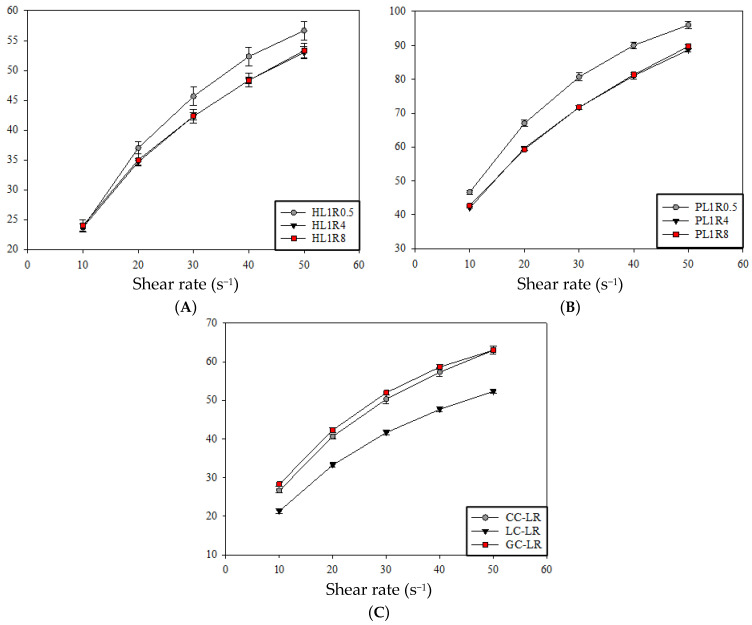
Shear stress—shear rate relationship plots: (**A**) Honey-like emulsion, (**B**) Pudding-like emulsion, (**C**) Calcium-modified emulsion. Error bars represent standard deviations (n = 3). Formulas are shown in Section 4.2.2.

The rheological analysis focused on characterizing the non-Newtonian behavior of thickened food emulsions. The Flow Behavior Index (*n*) is crucial for safe swallowing: a value of *n* < 1 indicates shear-thinning behavior; n = 1 indicates Newtonian behavior; and *n* > 1 indicates shear-thickening behavior. The degree of shear-thinning is inversely related to *n*, meaning a lower *n* value signifies stronger thinning. Foods with low *n* values can significantly reduce viscosity at the high shear rates in the pharynx, potentially increasing the risk of aspiration. Conversely, products with higher *n* values can maintain viscosity at these shear rates, supporting safer swallowing [26]. Additionally, the Consistency Coefficient (*K*) reflects viscosity, with a higher *K* indicating greater shear stress is required to sustain flow at a given shear rate. The Yield Stress (*σ*_0_) is the minimum stress required to initiate flow, which is vital for bolus manipulation and swallowing initiation [27,28]. Figure 2 visually shows the relationship between shear stress (*σ*) and shear rate for all food emulsions. Data were initially fitted to the Herschel–Bulkley model (Table A1). Although the R^2^ values were high (0.992–0.999), the calculated *σ*_0_ values were all negative, indicating that this model was unsuitable for describing the rheological properties of these emulsions. Therefore, the Power Law model was used to analyze *K* and *n* (R^2^ values ranging from 0.982 to 0.999), while the Casson model was used to determine *σ*_0_ (R^2^ values ranging from 0.970 to 0.992). This combination appropriately describes the fluid characteristics (Table 3). All samples showed n values between 0.434 and 0.534, confirming that all food emulsions formulated with LBG, RF, and MCT—regardless of calcium modification—are shear-thinning, non-Newtonian fluids, consistent with the apparent viscosity results at 50 s^−1^ and 10 s^−1^. The shear-thinning behavior mainly results from the disentanglement of LBG’s polysaccharide chains under shear [28,29]. Specific rheological traits were observed across groups: In the honey-like category, the HL1R8 formula exhibited the highest *K* (8.08 ± 0.576 Pa·s) and *σ*_0_ (9.45 ± 0.819 Pa), along with the lowest *n* (0.485 ± 0.0150). The pudding-like group’s PL1R0.5 emulsion showed the highest *K* (17.94 ± 0.327 Pa·s) and *σ*_0_ (21.30 ± 0.421 Pa), with the lowest (0.434 ± 0.0025). In the calcium-modified group, both the CC-LR (Calcium Carbonate) and GC-LR (Calcium Gluconate) formulas had higher *K* and *σ*_0_ values and lower n values compared to the HL1R0.5 control. Conversely, the LC-LR (calcium lactate) formula displayed lower *K* and *σ*_0_ and a higher *n*. Overall, pudding-like emulsions exhibited higher apparent viscosity (*K*) and *σ*_0_ but lower *n* than honey-like emulsions, a trend also observed in the calcium-modified groups. This indicates a positive relationship between apparent viscosity, *K*, and *σ*_0_, and an inverse relationship with *n*, suggesting that more viscous emulsions tend to demonstrate a greater degree of shear-thinning [27,29,30,31,32].

#### 2.1.5. Impact of Simulated SSF Buffer and α-SSF on Apparent Viscosity

This study used both SSF buffer (without α-amylase) and in vitro SSF with α-amylase (α-SSF) to accurately distinguish the effects of simple saliva dilution from those of salivary α-amylase on food emulsions.

**Figure 3:**
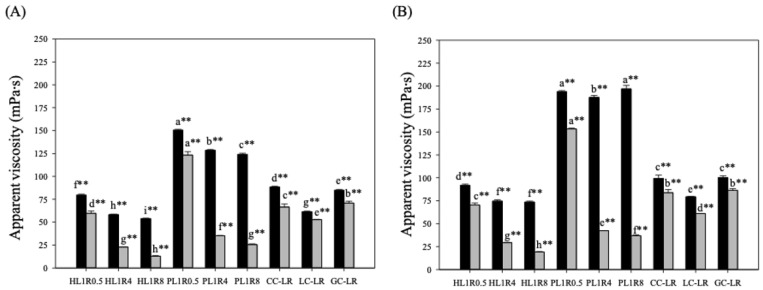
Apparent viscosity of food emulsions after adding SSF Buffer (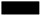) and α-SSF (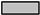): (**A**) 50 s^−1^ and (**B**) 10 s^−1^. Formula names are shown in Section 4.2.2. Error bars represent standard deviations (n = 3). The small letters in the same shear rate are significantly different (*p* < 0.05). ** indicates extremely significant difference (*p* < 0.01) between 50 s^−1^ and 10 s^−1^.

Regarding the dilutive effect of the SSF buffer, Figure 3A shows the apparent viscosity results at a shear rate of 50 s^−1^ after adding either the SSF buffer or the α-SSF. Compared with the initial viscosity before dilution, the viscosity of all samples decreased by more than 90%. Specifically, in the honey-like group, HL1R0.5 viscosity dropped by 92.40%, HL1R4 by 94.41%, and HL1R8 by 94.75%. In the pudding-like group, PL1R0.5 viscosity decreased by 91.69%. This drastic reduction is primarily attributed to the 1:1 (*w*:*w*) dilution ratio mandated by the INFOGEST protocol, which significantly alters the volume fraction of the polysaccharide network. While composition changes influence the degree of this drop, the impact of simple hydration remains the dominant factor in this initial viscosity loss.

The calcium-modified groups showed different protective effects against enzyme breakdown. While CC-LR and GC-LR kept higher viscosities and stronger barriers to α-amylase, the LC-LR group behaved differently. Although LC-LR had a relatively low degradation rate (14.16%), its initial apparent viscosity was much lower than that of the HL1R0.5 control. This lower starting point suggests that Calcium Lactate (LC) might alter the starch structure or disrupt the initial formation of the LBG-RF network, as seen in rice-based systems. Therefore, although CC and GC effectively increase both initial viscosity and enzyme resistance, LC mainly affects the matrix structure, resulting in less effective thickening for dysphagia management.

The addition of LC and GC significantly enhanced the resistance of the LBG-RF emulsions to enzymatic breakdown. This protective effect likely stems from the Ca^2+^ ions promoting a more robust starch-gum interpenetrating network, which physically hinders enzyme diffusion [8].

The effect of enzymatic degradation was assessed by comparing the viscosity of samples diluted with α-SSF to those diluted with SSF buffer (without α-amylase). The viscosity of PL1R0.5 decreased by 18.02% after α-SSF treatment compared to the buffer, indicating minimal impact from α-amylase degradation, while HL1R0.5’s viscosity dropped by 24.94%, making it the second lowest. Both PL1R0.5 and HL1R0.5 maintained their viscosity within the NDD nectar-thick liquid range (51–350 mPa·s). In contrast, HL1R4, HL1R8, PL1R4, and PL1R8 showed significant viscosity reductions of 60% to 79%, classifying them as NDD thin liquids (1–50 mPa·s). These findings demonstrate that increasing the proportion of locust bean gum (LBG) makes the food emulsion less susceptible to α-amylase degradation [25], which hydrolyzes starch, disrupts its structure, and lowers the sample’s viscosity [10]. A sudden drop in oral viscosity poses a major safety concern [9] in dysphagia management because of the risk of aspiration; therefore, the L1R4 and L1R8 food emulsions are less suitable for dysphagia patients compared to the L1R0.5 formula.

The calcium-modified groups demonstrated a protective effect against degradation: CC-LR viscosity dropped by 24.94%, GC-LR by 16.48%, and LC-LR by 14.16%. All three modified samples maintained their viscosity within the NDD nectar-thick liquid range (51–350 mPa·s), indicating that adding LC and GC helps prevent α-amylase degradation. Different calcium modifications may alter starch conformation, affecting not only the initial apparent viscosity but also subsequent α-amylase degradation [8]. Additionally, the dilution ratio recommended by the INFOGEST method had the most significant overall effect on reducing viscosity.

The apparent viscosity results at 10 s^−1^ (a shear rate relevant to oral perception) after adding SSF buffer or α-SSF are shown in Figure 3B. A significant reduction in viscosity due to dilution was observed across all samples following SSF buffer treatment: for the honey-like group, HL1R0.5 viscosity decreased by 95.82%, HL1R4 by 96.68%, and HL1R8 by 96.83%, with HL1R0.5 remaining the most viscous. In the pudding-like group, PL1R0.5 (95.54%) and PL1R8 (95.05%) exhibited higher viscosity values than PL1R4 (95.33%). In the calcium-modified group, CC-LR (96.25%) and GC-LR (96.17%) maintained higher viscosities than LC-LR (96.20%). Notably, the honey-like samples HL1R4 and HL1R8, the pudding-like samples PL1R0.5 and PL1R8, and the calcium-modified samples CC-LR and GC-LR showed no significant difference in perceived oral viscosity after SSF buffer dilution.

Consistent with the 50 s^−1^ results, adding α-SSF led to a more noticeable reduction at 10 s^−1^, and differences were seen across most food emulsions, except for CC-LR and GC-LR. In the honey-like group, HL1R0.5 exhibited the least α-amylase degradation, with viscosity decreasing by 23.39% compared to the SSF buffer. In the pudding-like group, PL1R0.5 also showed the smallest degradation, with a 20.98% decrease in viscosity. These findings confirm that α-amylase has a stronger hydrolytic effect on the L1R8 formula. When comparing the calcium-modified groups to HL1R0.5, CC-LR and GC-LR displayed higher viscosities, with reductions of 15.69% and 13.76%, respectively. Conversely, LC-LR had a lower viscosity, with a 23.06% reduction. Furthermore, text CC-LR and GC-LR showed no significant differences in perceived oral viscosity. Overall, these results indicate that adding CC, LC, and GC helps decrease the degradation of starch-containing thickening emulsions by α-amylase, especially regarding perceived oral viscosity.

#### 2.1.6. Rheological Properties After Salivary Treatment

This study selected HL1R0.5, PL1R0.5, and the calcium-modified groups for further rheological analysis because these formulas showed less viscosity reduction due to salivary dilution and degradation.

**Figure 4:**
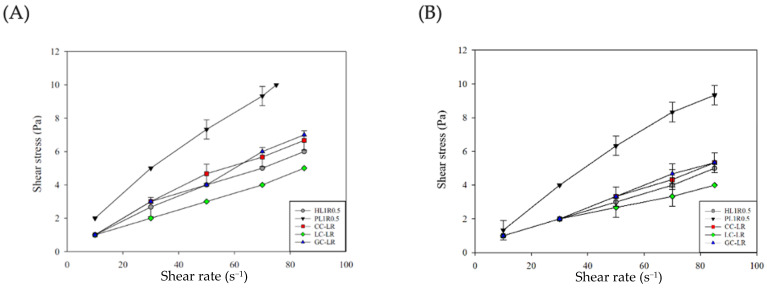
Shear Stress-shear rate curves of HL1R0.5, PL1R0.5, CC-LR, LC-LR, and GC-LR after adding SSF buffer (**A**) and α-SSF (**B**). Formula abbreviations are shown in Section 4.2.2. Error bars represent standard deviations (n = 3).

Figure 4A shows the shear stress (*σ*) versus shear rate gamma point curves measured after adding buffer. Due to the lower dilution, the measured shear stress for PL1R0.5 exceeded the detection range of the Spindle TL6 probe at 85 s^−1^. Therefore, the PL1R0.5 sample treated with SSF buffer was analyzed using shear stress data at 10, 30, 50, 70, and 75 s^−1^. Figure 4B displays the shear stress-shear rate curves of α-SSF. Compared to the food emulsions before α-SSF treatment (Figure 4A), the shear stress of all food emulsions decreased significantly after treatment with either SSF buffer or α-SSF, and the curves became more linear.

The samples treated with SSF buffer and α-SSF were analyzed using the Herschel–Bulkley model to determine the flow behavior index (*n*), the consistency coefficient (*K*), and the yield stress (*σ*_0_). Data are shown in Table A1. Likewise, the results for untreated food emulsions showed that, although the R^2^ values were high (ranging from 0.966 to 0.999 for SSF buffer and 0.921 to 0.999 for α-SSF), the *σ*_0_ values for HL1R0.5, PL1R0.5, and CC-LR after SSF buffer addition, and for PL1R0.5 after α-SSF addition, were negative.

Therefore, the Power-law model was used to analyze the *n* and *K* values, while the Casson model was applied to analyze *σ*_0_. The results are summarized in Table 3. The R^2^ values for the Power-Law analysis ranged from 0.962 to 0.991 (SSF buffer) and from 0.919 to 0.991 (α-SSF). The R^2^ values for the Casson model analysis ranged from 0.969 to 0.995 (SSF buffer) and from 0.936 to 0.995 (α-SSF). This confirms that combining the Power-law and Casson models effectively describes the fluid properties of the food emulsions after treatment with the SSF buffer or α-SSF. Table 3 Power law model and Casson model fit results of HL1R0.5, PL1R0.5, CC-LR, LC-LR, and GC-LR after adding SSF Buffer and α-SSF. While the effect of α-SSF degradation on rheology depends on the formulation, samples treated with α-amylase consistently showed decreases in *K* values and increases in *n* values for most food emulsions, except LC-LR. This variability indicates that the specific interaction between the polysaccharide network and added calcium ions influences the extent of structural breakdown during the oral phase.

Regarding the effect of SSF buffer dilution on rheology, after adding SSF buffer, all samples showed decreases in both *K* and *σ*_0_, while *n* increased. This indicates that the food emulsions become more like Newtonian fluids after SSF buffer treatment, mainly due to simple dilution. Consistent with the untreated samples, the more viscous PL1R0.5 had higher *K* and *σ*_0_ values than HL1R0.5. Among the calcium-modified groups, LC-LR had a lower *K* value than HL1R0.5, while GC-LR had a higher *n*.

While the effect of α-SSF degradation on rheology is subjective, results for samples treated with α-SSF (containing α-amylase) showed a consistent decrease in *K* values and an increase in *n* values across all food emulsions, except LC-LR. The PL1R0.5 formulation maintained a higher *K* value than HL1R0.5, indicating greater viscosity (Table 3). In the calcium-modified groups, LC-LR exhibited higher *K* and *σ*_0_ values and a lower *n* value compared to HL1R0.5.

The overall results show that the flow behavior of food emulsions is significantly influenced by salivary dilution, which decreases *K* and *σ*_0_ and increases *n*. Further degradation by α-amylase results in a continuous decrease in *K* values and a steady increase in *n* values for most emulsions, except for LC-LR. Notably, adding calcium lactate (LC) resulted in a stronger shear-thinning behavior in the food emulsion after α-amylase degradation.

### 2.2. IDDSI Classification

#### 2.2.1. Syringe Flow Test

**Figure 5:**
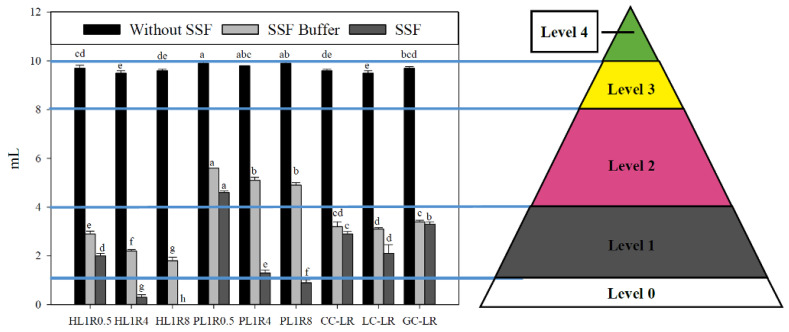
IDDSI syringe flow test results of native food emulsions without SSF, with SSF Buffer, and with α-SSF. Formula names are shown in Section 4.2.2. Error bars represent standard deviations (n = 3). The lowercase letters in the same bar are significantly different (*p* < 0.05).

The IDDSI flow test classifies thickened liquids into five levels based on the residual volume in a 10 mL syringe after 10 s, using a standard 10 mL syringe: Level 0 (thin, 0–1.0 mL), Level 1 (slightly thick, 1.0–4.0 mL), Level 2 (mildly thick, 4.0–8.0 mL), Level 3 (moderately thick, 8.0–10.0 mL), and Level 4 (extremely thick, mL, completely non-flowing). The results are illustrated in Figure 5.

All samples not treated with the SSF buffer showed a residual volume over 8 mL after the 10-s test. When comparing the pudding-like samples (PL1R0.5, PL1R4, and PL1R8) with their honey-like counterparts (HL1R0.5, HL1R4, and HL1R8), the pudding-like samples consistently had higher residual volumes. Among the calcium-modified groups, the LC-LR sample had a lower residual volume compared to HL1R0.5.

Although all samples were classified as IDDSI Level 3 (moderately thick) based on the syringe flow test, they corresponded to the honey-thick and pudding-thick categories under NDD standards. Additionally, minor variations in residual volume were observed even within the same NDD classification, and the results did not perfectly match viscosity measurements. For example, before α-SSF addition (Table 2), CC-LR had an apparent viscosity of 1246.37 ± 10.017 mPa·s at 50 s^−1^, which exceeded HL1R0.5’s 1046.27 ± 15.542 mPa·s. However, the syringe flow test showed similar residual volumes of 9.6 ± 0.06 mL and 9.7 ± 0.12 mL, respectively. Research by Gamonpilas et al. [33] indicated that the shear rate in the syringe flow test is not constant, and extensional flow significantly influences residual volume, which is strongly affected by the structural morphology of each liquid. Therefore, when preparing products for dysphagia patients, it is important to consider not only differences among classification standards but also the disparities resulting from the varying compositions of the food products.

The syringe flow test was also conducted on samples treated with SSF buffer (to simulate dilution) and α-SSF (to simulate both dilution and degradation) to mimic oral processing. Notably, after adding the SSF buffer, all pudding-like samples were classified as IDDSI Level 2 (Mildly Thick). The PL1R0.5 sample had the highest residual volume at 5.6 ± 0.01 mL, which was significantly different from the others (*p* < 0.05). The honey-like group showed a similar pattern: HL1R0.5 had the highest residual volume at 2.9 ± 0.12 mL, followed by HL1R4, with HL1R8 having the lowest, and there were significant differences among all three (*p* < 0.05). In the calcium-modified group, CC-LR, LC-LR, and GC-LR all exhibited higher syringe residual volumes than the HL1R0.5 control. This suggests that adding CC, LC, and GC helps keep the syringe residual volume of the food emulsion stable after SSF buffer treatment. Interestingly, this finding conflicts with previous viscosity measurements, where adding CC, LC, and GC increased the dilution effect of SSF buffer on apparent viscosity. This discrepancy highlights differences between various classification standards and measurement methods. After α-SSF treatment, viscosity significantly decreased, resulting in lower IDDSI levels: in the honey-like group, HL1R0.5 shifted to IDDSI Level 1 (slightly thick), while HL1R4 and HL1R8 further decreased to IDDSI Level 0 (thin), with significant differences among the three (*p* < 0.05). In the pudding-like group, PL1R0.5 dropped to IDDSI Level 2 (mildly thick), PL1R4 to IDDSI Level 1, and PL1R8 to IDDSI Level 0, with notable differences among the groups (*p* < 0.05). In the calcium-modified group, CC-LR and GC-LR showed higher syringe residual volumes than HL1R0.5. This correlates with viscosity measurements, indicating that adding CC and GC helps prevent α-amylase-induced breakdown of the food emulsion. Furthermore, we observed that syringe flow results did not perfectly mirror apparent viscosity. This is consistent with previous research indicating that the IDDSI syringe test is heavily influenced by extensional flow and the liquid’s unique structural morphology, whereas rotational viscosity accounts only for shear resistance [33].

#### 2.2.2. Fork Drip Test

The manual fork drip and spoon tilt tests are subjective and qualitative methods that lack a quantitative component for measuring food texture. Because they do not involve rigorous texture analysis or expert interpretation, these assessments are often considered impractical for precise measurement. To improve accuracy and generate measurable, comparable data, additional research using instrumented methods across various food emulsions with different compositions is necessary. Accordingly, the study employed a texture analyzer with the IDDSI-F (A/IDDSI-F) and IDDSI-SC (A/IDDSI-SC) probes to mimic manual utensil analysis. The probe was programmed to scoop to a consistent depth below the liquid surface, lift above the surface, and hold at that depth for 5 s. The initial weight and the remaining weight after 5 s were calculated using Exponent software (Version 6). Combining the A/IDDSI-F, A/IDDSI-SC, and manual utensil analysis aimed to improve the accuracy of food emulsion assessment and enable quantification of the IDDSI classification. According to the IDDSI classification, the fork drip test involves immersing a four-tine fork with 4 mm space between the fork tines into the test liquid, then removing it and observing the result. Samples that remain piled on top of the fork are classified as Level 4, while those that slowly drip through the tins are classified as Level 3.

**Figure A2:**
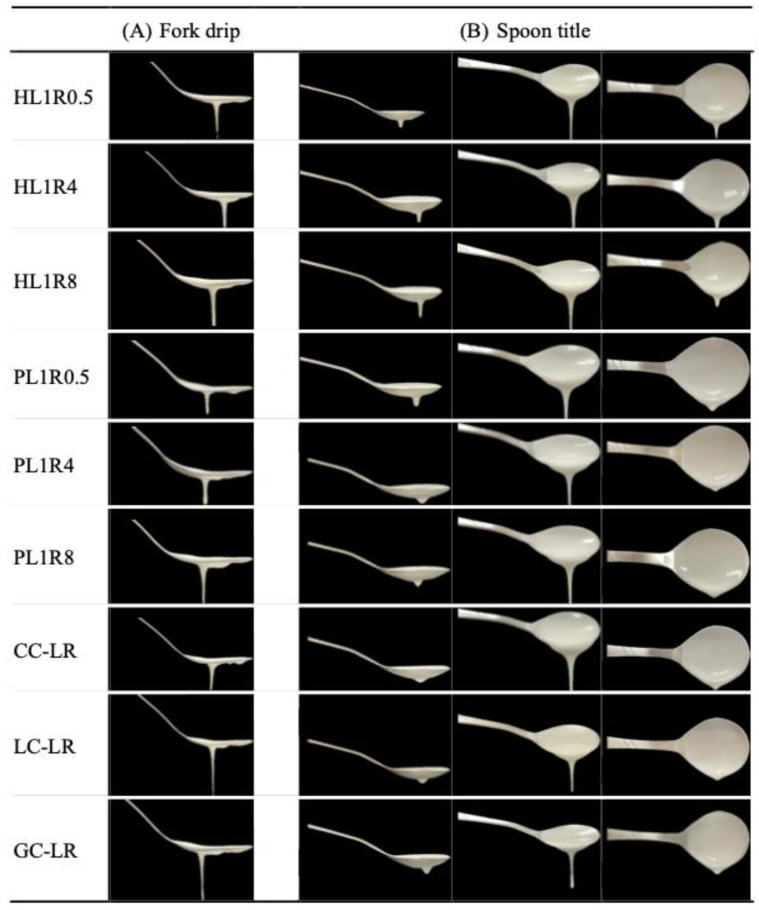
IDDSI fork-drip test and spoon-tilt test. Formulas are shown in Table 6.

In Figure A2, all samples gradually dripped through the fork tines, and no sample formed a mound on top of the fork. Therefore, based on manual observation, all samples were classified as IDDSI Level 3 liquids. The results for the instrument-assisted A/IDDSI-F test are shown in Table 4.

While the fork drip test and spoon tilt test effectively established the baseline classification of all native emulsions as IDDSI Level 3, their qualitative nature makes them less suitable for assessing low-viscosity liquids produced by α-amylase degradation. Therefore, the syringe flow test was used as the primary quantitative measure to monitor changes after salivary treatment. The syringe test is specifically designed to distinguish between the lower IDDSI levels (0–3) that occur when the polysaccharide network is compromised, while the fork drip and spoon tilt tests are better suited for thicker, semi-solid boluses.

It was observed that the initial weights of the honey-like emulsions (HL1R0.5, HL1R4, and HL1R8) were very similar, ranging from 3.968 to 4.288 g, with no significant differences. However, when it thickened to a pudding-like consistency, the initial weight increased significantly, ranging from 7.418 to 10.010 g. Among the pudding-like samples, PL1R0.5 had the highest initial weight, followed by PL1R4, and PL1R8 had the lowest, with significant differences among the three samples (*p* < 0.05). Comparing the initial weights of the calcium-modified groups with HL1R0.5, the addition of calcium generally increased the initial weight of the food emulsion. CC-LR (5.273 ± 0.2948 g) and GC-LR (5.384 ± 0.1202 g) showed the highest initial weights, followed by LC-LR (4.841 ± 0.3113 g).

According to the IDDSI classification, the fork drip test involves immersing a four-tine fork with 4 mm space between the fork tines into the test liquid, then removing it and observing the result. Samples that remain piled on top of the fork are classified as Level 4, while those that slowly drip through the tins are classified as Level 3.

In Figure A2, all samples gradually dripped through the fork tines, and no sample formed a mound on top of the fork. Therefore, based on manual observation, all samples were classified as IDDSI Level 3 liquids. The results for the instrument-assisted A/IDDSI-F test are shown in Table 4. It was observed that the initial weights of the honey-like emulsions (HL1R0.5, HL1R4, and HL1R8) were very similar, ranging from 3.968 to 4.288 g, with no significant differences. However, when thickened to a pudding-like consistency, the initial weight increased significantly, ranging from 7.418 to 10.010 g. Among the pudding-like samples, PL1R0.5 had the highest initial weight, followed by PL1R4, and PL1R8 had the lowest, with significant differences among the three samples (*p* < 0.05). Comparing the initial weights of the calcium-modified groups with HL1R0.5, the addition of calcium generally increased the initial weight of the food emulsion. CC-LR (5.273 ± 0.2948 g) and GC-LR (5.384 ± 0.1202 g) showed the highest initial weights, followed by LC-LR (4.841 ± 0.3113 g).

#### 2.2.3. Spoon Tilt Test

For Level 4 (Extremely Thick) and Level 5 (minced and moist) foods, the liquid should maintain its shape on the spoon. Level 3 (moderately thick) liquids, however, cannot keep their shape and slide easily off the spoon when tilted, leaving only a small amount of residue. This feature helps the food be easily swallowed without sticking to the tongue or the pharynx; on the other hand, thicker foods may increase the risk of aspiration [34].

As shown in Figure A2, all samples failed to maintain their shape on the spoon and easily slid off when tilted, with only a small amount remaining on the bottom surface. Therefore, according to the IDDSI manual, all samples were classified as IDDSI Level 3: Moderately Thick liquids. The results for the instrument-assisted A/IDDSI-SC test are shown in Table 4. In the honey-like group, the initial weights of HL1R0.5 (7.344 ± 0.3187 g) and HL1R8 (7.167 ± 0.2209 g) were very similar, while HL1R4 had a lower initial weight (6.621 ± 0.5344 g). When thickened to a pudding-like consistency, the initial weight increased significantly, ranging from 9.585 to 10.303 g. Notably, PL1R4 had the highest initial weight, with a significant difference compared to PL1R0.5 and PL1R8 (*p* < 0.05). Compared to HL1R0.5, the addition of calcium generally increased the initial weight of the food emulsion in the calcium-modified groups, with CC-LR, LC-LR, and GC-LR having initial weights between 8.083 and 8.376 g.

The A/IDDSI-SC 5-s weight results (Table 4) showed that HL1R0.5 had the highest 5-s weight (1.598 ± 0.3050 g) in the honey-like group, followed by HL1R4 and HL1R8 (0.875 to 1.090 g). A similar trend was observed for the pudding-like group: PL1R0.5 had the highest 5-s weight (1.827 ± 0.1250 g), followed by PL1R4 and PL1R8 (1.327 to 1.376 g). Compared with HL1R0.5, the addition of all three calcium sources significantly reduced the 5-s weight of the food emulsion (*p* < 0.05).

Based on the combined manual spoon tilt test and A/IDDSI-SC analysis, an initial weight of 6.621–10.303 g and a 5-s weight of 0.875–1.827 g classify the product as IDDSI Level 3 liquid. To further categorize according to NDD standards, considering the calcium-modified samples were honey-thick and the pudding-like samples were near the viscosity threshold, the following threshold criteria were established for the spoon tilt est results.

### 2.3. Texture Profile Analysis (TPA)

Thickened liquids are often used in the care of dysphagia patients because their slower flow during oral and pharyngeal swallowing provides better control, thereby improving swallowing safety. The safest and simplest way for these patients to consume food involves items that do not require chewing or changes in bolus consistency. The bolus should be soft (low hardness), easily form cohesive lumps in the mouth and pharynx (high cohesiveness), and be able to be propelled without sticking to the mucosa (low adhesiveness) [35]. Table 5 presents the results of the texture profile analysis (TPA) for different food emulsions. While rheological measurements (Table 2) describe the flow resistance and apparent viscosity of the samples, TPA provides insights into the mechanical textural parameters—Hardness, Adhesiveness, Cohesiveness, and Gumminess—that are essential for evaluating swallowing safety. These parameters reflect how the bolus is manipulated during the oral and pharyngeal phases, where low hardness and high cohesiveness are prioritized to prevent aspiration.

The hardness results showed that in the honey-like group, HL1R0.5 had the highest hardness, followed by HL1R4, with HL1R8 being the lowest. When the samples were thickened into a pudding-like matrix, PL1R4 exhibited the highest hardness, followed by PL1R0.5, and PL1R8 showed a slight decrease but no significant difference. Compared to HL1R0.5, all three calcium sources significantly reduced the sample hardness in the calcium-modified groups, with GC-LR showing the highest hardness, followed by CC-LR and LC-LR. The increase in hardness observed in some gum-starch binary gels can be attributed to the formation of hydrogels by galactomannans in the aqueous solution [36]. Compared to the HL1R0.5 control, all three calcium sources significantly reduced the sample hardness. These decreases likely result from calcium ions interacting with the polysaccharide components; specifically, calcium can alter starch conformation and potentially disrupt the extensive hydrogen bonds and molecular chain entanglements characteristic of the LBG-RF network. By interfering with these cross-linking points, calcium prevents the formation of a rigid three-dimensional matrix, resulting in a softer and more flexible food bolus. This reduction in hardness benefits individuals with dysphagia by making bolus formation easier and reducing the muscular effort required for safe swallowing.

Gumminess, which is calculated as the product of hardness and cohesiveness, relates to the force needed to break down semi-solid food into a soft, easy-to-swallow bolus; therefore, its trend agrees with that of hardness. Foods for dysphagia patients should have low hardness and gumminess to reduce the risk of choking. Notably, when comparing PL1R8 (390.023 ± 14.2817) and HL1R0.5 (403.596 ± 5.3864), PL1R8 exhibited lower gumminess due to its lower cohesiveness.

When comparing adhesiveness across samples, HL1R0.5 showed the highest in the honey-like group, followed by HL1R4, with HL1R8 being the lowest. When thickened into a pudding-like matrix, PL1R4 exhibited the highest adhesiveness. Meanwhile, PL1R0.5 showed a slight decrease without a significant difference, while PL1R8 had the lowest adhesiveness, with a significant difference compared to PL1R0.5 and PL1R4. Overall, pudding-like food emulsions had higher adhesiveness than honey-like emulsions. In the calcium-modified groups, all three modifications reduced the samples’ adhesiveness compared to HL1R0.5, with CC-LR showing the highest, followed by LC-LR and GC-LR. Adhesiveness may be related to the residual amount observed in the spoon tilt test. However, the results from the A/IDDSI-SC 5-s weight test (Table 4) did not follow the same trend. This discrepancy could be due to differences in probe material: the TPA used an acrylic cylindrical probe, while the A/IDDSI-SC test used a metal spoon. Previous studies [37] have indicated that the surface properties of the probe can influence adhesiveness measurements, which could explain the differences between the results of the adhesiveness and Spoon tilt tests.

The cohesiveness results in Table 5 showed that HL1R0.5 had the highest cohesiveness in the honey-like group, while HL1R8 was the lowest. A similar pattern was observed in the pudding-like matrix, where PL1R0.5 demonstrated the highest cohesiveness. Overall, the pudding-like food emulsions generally exhibited greater cohesiveness than the honey-like food emulsions, as seen in the comparison between PL1R4 (0.99 ± 0.01) and HL1R4 (0.96 ± 0.01). This higher cohesiveness in the pudding-like group indicates a stronger internal structure, which helps maintain bolus integrity during the pharyngeal phase of swallowing.

In the calcium-modified groups, adding any of the three calcium sources reduced the sample’s cohesiveness compared to HL1R0.5. Notably, LC-LR had the lowest cohesiveness, with a significant difference from HL1R0.5 (*p* < 0.05). This pattern is similar to the difference observed between starch-based and gum-based thickeners, where starch-based thickeners tend to have significantly lower cohesiveness than gum-based ones [38,39].

The experimental results show that HL1R0.5, PL1R0.5, PL1R4, PL1R8, and GC-LR food emulsions meet the Japanese Dysphagia Food Approval Standard III, which requires hardness to be between 300 and 20,000 N/m^2^ and adhesiveness to be below 1500 J/m^3^. The hydrocolloids used in this study can entangle or interact with starch molecules, causing the gel structure to reorganize and form a stable starch gel network [40].

The food emulsions were semi-solid before α-SSF was added, but they became more liquid afterward. While the initial texture profile analysis results (Table 5) characterize the baseline mechanical properties of the semi-solid emulsions, the structural changes following α-SSF addition were monitored through rheological and syringe flow assessments rather than TPA. The rapid liquefaction caused by α-amylase resulted in a loss of the bolus-like consistency required for instrumental texture analysis using a compression probe. Therefore, the protective effect of calcium against enzymatic degradation is primarily reflected in the maintained apparent viscosity (Table 2) and the residual volume results from the syringe flow test (Section 2.2.1), rather than changes in TPA parameters.

A previous study [41] has demonstrated that, during TPA, when a probe compresses liquid food, the measured force gradually increases with compression and then decreases as the probe stops or pulls back. This suggests that the measured force may not accurately reflect actual hardness. Similarly, adhesiveness is influenced by the speed and extent of compression, as well as by the probe’s surface characteristics. When testing liquid foods, noise from the probe reversing direction and the unique compression behavior of liquids can usually be ignored for samples with comparably great magnitudes. However, for samples with smaller values, this noise can account for up to half of the maximum measurement, making it a significant source of error. Therefore, practitioners should be aware of these potential errors when performing TPA on liquid foods and consider alternative testing methods or improved conditions to reduce noise and inaccuracies [42].

### 2.4. Microstructural Analysis

Emulsifiers work by adsorbing at the interface, lowering the interfacial tension between the two phases, and increasing steric hindrance and electrostatic repulsion between droplets. This process prevents droplet coalescence, thereby reducing droplet size and improving emulsion stability [43].

#### 2.4.1. Honey-like Food Emulsions

**Figure 6:**
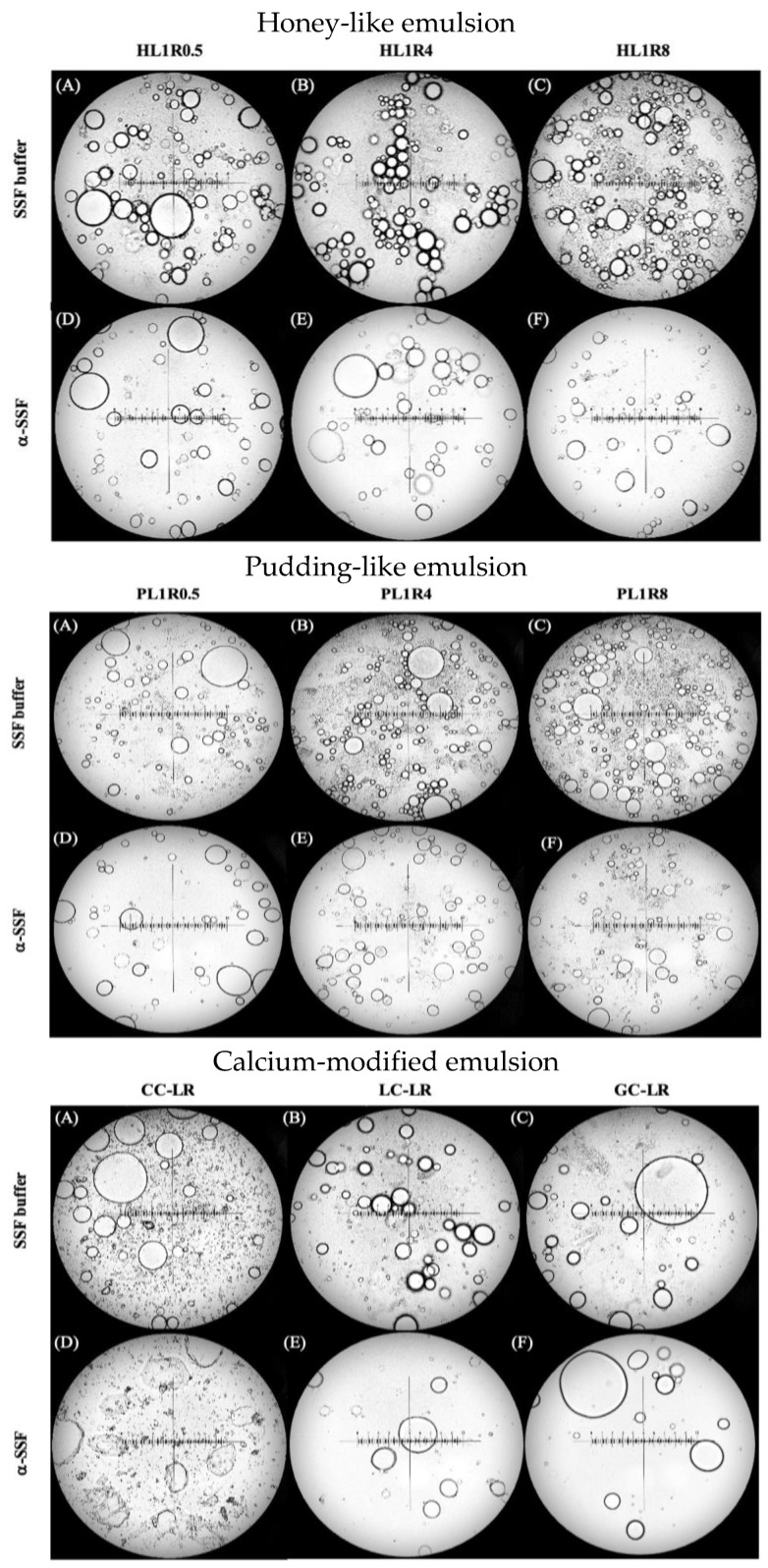
Microscopic images of honey-like, pudding-like, and calcium-modified emulsions: (**A**–**C**) SSF buffer, (**D**–**F**) α-SSF. Formulas are shown in Section 4.2.2.

Figure 6 displays the photomicrographs of the HL1R0.5, HL1R4, and HL1R8 food emulsions. For the SSF buffer, the untreated samples showed that as the proportion of pre-gelatinized rice flour (RF) increased, droplet size gradually decreased, and the particle size distribution became more uniform and compact, with more aggregated starch granules observed. These observations suggest that pre-gelatinized rice flour has effective emulsifying properties. This aligns with previous studies where confocal laser scanning microscopy demonstrated that starch in an oil-in-water emulsion (made of 5.0% peanut oil and 95.0% starch supernatant) concentrates at the water-oil interface, indicating that amylose can act as an emulsifier, giving starch dual affinity for water and oil, which helps stabilize the interface [44]. Similarly, research on emulsions made from quinoa, waxy barley, and oat starches found that small amounts of starch granules attach to the droplet surface. In contrast, most granules remain free, forming clusters around the main droplets or aggregating in the continuous phase. In these cases, droplet size decreases as starch content increases [45].

In samples treated with α-SSF, the particle size distribution appeared more dispersed, and the droplet size increased compared to untreated emulsions. Additionally, a decrease in the number of aggregated starch granules was observed, indicating that the structure of the pre-gelatinized rice flour had broken down. This aligns with earlier research [46] on meat sauces containing 15% fat and 3.75% starch, in which fat droplets were visible after simulated chewing. In contrast, starch granules were no longer visible, likely due to amylase digestion. The combined effects of SSF buffer dilution and α-amylase hydrolysis of α-1-4 glycosidic bonds during oral processing disrupted the emulsion’s interfacial layer, reducing aggregated starch granules and droplet coalescence. Viscosity measurements support this microstructural breakdown [47].

#### 2.4.2. Pudding-like Food Emulsions

Figure 6 shows the photomicrographs of the PL1R0.5, PL1R4, and PL1R8 food emulsions. A similar trend to that of the honey-like food emulsions was observed: before α-SSF addition, increasing the amount of pre-gelatinized rice flour resulted in smaller droplet sizes, a more uniform, denser particle-size distribution, and a higher number of aggregated starch granules. After α-SSF treatment, the particle size distribution became more dispersed, and the number of aggregated starch granules decreased compared to the untreated emulsions.

Furthermore, a comparison between the pudding-like and honey-like groups after α-SSF treatment showed an increase in aggregated starch granules in PL1R4 and PL1R8. This indicates that the 2-min in vitro oral digestion process was not enough to fully break down the pre-gelatinized rice flour structure in these higher-viscosity formulas. Therefore, when preparing products for dysphagia patients, it is important to consider the enzyme kinetics of α-amylase on starch to ensure sufficient carbohydrate sources are available for nutritional balance.

#### 2.4.3. Calcium-Modified Food Emulsions

The microstructural results for calcium-modified food emulsions are shown in Figure 6. In the untreated CC-LR emulsion (SSF buffer), CC particles were clearly visible. Even after α-SSF treatment, CC particles remained, partially obscuring the observable droplets and making it difficult to assess droplet size and distribution. This indicates that neither the α-SSF dilution effect nor α-amylase degradation significantly decreased the CC particle content.

Comparing LC-LR with HL1R0.5, LC-LR showed smaller droplet sizes and a broader particle size distribution. After α-SSF treatment, the droplet size increased, and the distribution became even wider. In contrast, GC-LR had larger droplet sizes and a broader particle size distribution than HL1R0.5. Following α-SSF treatment, the droplet size increased again, and the distribution broadened, similar to the change observed in the LC-LR sample after α-SSF treatment.

#### 2.4.4. Microstructural Differences Correlate with Sensory Attributes

The microstructural features of food emulsions form the physical basis for their perceived sensory qualities. As the proportion of pre-gelatinized rice flour (RF) increases, the droplet size decreases, and the distribution becomes more uniform. In food emulsions, smaller oil droplets (typically less than 10 μm) are known to enhance perceptions of ‘creaminess’ and ‘smoothness’ by providing a larger surface area for interaction with oral receptors and reducing friction [46]. However, the increased presence of aggregated starch granules observed in the L1R8 formulations may cause a slight ‘granularity’ or ‘pastiness’. When treated with α-amylase, the structural breakdown—characterized by increased droplet coalescence and fewer visible starch aggregates—correlates with a rapid decrease in perceived viscosity (oral thinning), which is a critical safety factor for dysphagia patients. The preservation of more intact structures in the calcium-modified GC-LR group after α-SSF treatment likely supports a more consistent and safer sensory experience during the oral phase [9].

### 2.5. Emulsion Stability

**Figure 7:**
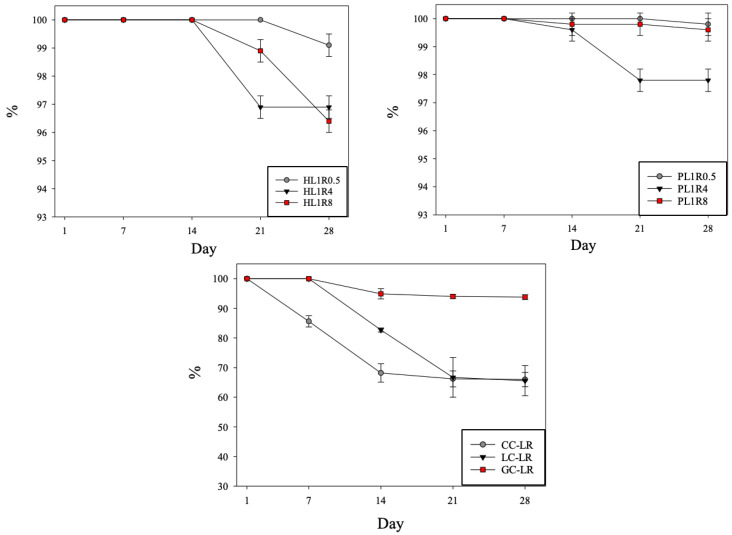
Stability trends of food emulsions. Error bars represent standard deviations (n = 3). Formulas are shown in Section 4.2.2.

The storage stability of various food emulsions was assessed over 28 days at 4 °C. Figure 7 shows the trend of stability changes over time.

#### 2.5.1. Stability of LBG-RF Ratio Groups

The results showed differences in stability among the honey-like samples: the stability of HL1R0.5 remained steady for 21 days, then slightly dropped to 99.11 ± 0.38% by Day 28. HL1R4 and HL1R8 remained stable for 14 days, then decreased to 96.89 ± 0.38% and 98.89 ± 0.38%, respectively, by Day 21. By Day 28, HL1R4 had stabilized, while HL1R8 continued to decline to 96.44 ± 0.38%. This indicates that the HL1R0.5 formula had the best storage stability among the honey-like samples.

When the emulsion thickened to a pudding-like consistency, its storage stability generally improved because the aqueous phase surrounding the oil droplets became more viscous [48]. The stability of PL1R0.5 remained steady for 21 days, only slightly decreasing to 99.78 ± 0.38% on Day 28. PL1R4 and PL1R8 showed a small decline in stability by Day 14, reaching 99.56 ± 0.38% and 99.78 ± 0.38%, respectively. By Day 28, PL1R8 was slightly lower at 99.56 ± 0.38%, while PL1R4 continued to decrease to 97.78 ± 0.38%, indicating it was the most unstable in the pudding-like emulsions. It is hypothesized that the LBG and RF did not reach a stable state at this specific ratio, leading to RF sedimentation and MCT separation.

#### 2.5.2. Role of Viscosity and Microstructure

Although droplet size increased with higher LBG and RF ratios (as shown in the Section 2.4), the emulsions remained stable during storage. Therefore, small droplet size does not always guarantee stability; other factors, such as rheology and flocculation potential, must also be considered [49]. The stability of the oil droplets primarily results from the high viscosity of the aqueous phase, which is generated by the network formed by the LBG-RF complex. This increased viscosity prevents droplets from colliding, thereby reducing flocculation and coalescence. Previous research has demonstrated that polysaccharide dispersions create a sufficiently viscous continuous phase that limits oil droplet movement, flocculation, and coalescence, thus decreasing droplet mobility and collision frequency [48].

#### 2.5.3. Effect of Calcium Modification

When comparing the calcium-modified groups with HL1R0.5, calcium addition generally decreased the stability of the food emulsion. LC-LR and CC-LR showed the lowest storage stability on Day 28, while GC-LR maintained the highest stability among the calcium-modified samples.

## 3. Conclusions

This study successfully developed a series of calcium-enriched food emulsions using a complex of Locust Bean Gum (LBG) and Pre-gelatinized Rice Flour (RF). The L1R0.5 ratio proved to be the most efficient formulation, requiring the lowest concentration to achieve the target viscosities. Notably, this ratio demonstrated superior resistance to salivary α-amylase; after treatment with α-SSF, the HL1R0.5 emulsion maintained 75.06% of its apparent viscosity at 50 s^−1^, remaining within the NDD nectar-thick range (51–350 mPa·s). In contrast, higher RF ratios (L1R4 and L1R8) experienced significant viscosity loss of 60% to 79%, dropping into the NDD thin liquid category. The addition of organic calcium sources further improved stability; calcium gluconate (GC) and calcium lactate (LC) reduced enzymatic degradation to as little as 14.16–16.48%. These findings imply that food manufacturers can use L1R0.5 as a stable base for energy-dense, 10% MCT-fortified diets that do not compromise safety during the oral phase. While this study provides a quantitative link between rheology and the IDDSI framework, future research should examine tribological properties to better understand oral lubrication and “mouthfeel.” Additionally, long-term clinical trials are needed to assess the impact of these emulsions on the nutritional status and swallowing safety in elderly patients with chronic dysphagia.

## 4. Materials and Methods

### 4.1. Materials and Reagents

All specialized food ingredients and additives were carefully sourced from reputable suppliers mainly in Taiwan, with specified material origins to ensure quality and consistency.

Pre-gelatinized rice flour was originally sourced from EGO International Co., Ltd. (Taipei, Taiwan), with origins in Croatia. The thickening agent, locust bean gum (Lot No. 2330378, Swiss origin), along with two calcium sources—calcium carbonate (Lot No. 23.08.03T, Japanese origin) and calcium lactate (Lot No. G-2304-364, Belgian origin)—were all supplied by Jen Fong Co., Ltd. (Taipei, Taiwan). Medium-chain triglycerides (MCTs) (Lot No. 7114434068) were obtained from Cheng Ting Enterprise Co., Ltd. (Taipei, Taiwan), with Malaysian origin. In contrast, calcium gluconate (Lot No. 20231103, Chinese origin) was acquired from Yuan Hong Herbal Science & Research Co., Ltd. (Taichung, Taiwan). Additionally, a variety of high-purity chemical reagents was used to prepare buffer solutions and enzymatic assays, including potassium chloride (KCl), dipotassium phosphate (K_2_HPO_4_), and sodium bicarbonate (NaHCO_3_), supplied by Honeywell Specialty Chemicals Seelze GmbH (Seelze, Germany). Magnesium chloride hexahydrate (MgCl_2_·6(H_2_O)) was obtained from Avantor Performance Materials, LLC (Radnor, PA, USA), while ammonium carbonate ((NH_4_)_2_CO_3_) and sodium hydroxide (NaOH) were supplied by UniRegion Bio-Tech (Frankfurt, Germany). Hydrochloric acid (HCl), calcium chloride dihydrate (CaCl_2_·2H_2_O), and α-amylase (Type VI-B) were purchased from Sigma-Aldrich Laborchemikalien GmbH (Seelze, Germany). All chemical reagents, including salts and acids, were of ACS reagent grade, and the α-amylase used had a guaranteed purity of ≥5 units/mg solid.

### 4.2. Experimental Methods

#### 4.2.1. Preparation of LBG-RF Complex Thickener and Calcium-Fortified Formulas

Thickening liquids were prepared to achieve a range of viscosities, from honey-like to pudding-like, based on the desired level of thickening. A specific amount of Locust Bean Gum (LBG) and pre-gelatinized rice flour (RF) was gradually mixed into 100 g of deionized water. For the calcium-modified groups, the calcium source (with different absorption rates) was first evenly dispersed in deionized water, and then the LBG-RF powder mixture was added.

The LBG-RF mixture was heated and stirred on a magnetic hot plate at 80 °C for 1 h to ensure complete dissolution and hydration of the polysaccharides. This protocol was established based on preliminary optimization tests. For instance, testing at 75 °C for 90 min resulted in incomplete hydration, such that the resulting matrix could not pass through an 80-mesh strainer without external pressure. Selecting 80 °C aligns with the requirement that LBG achieve optimal solubility upon heating. After this optimized heating phase, samples were cooled to room temperature before the incorporation of medium-chain triglycerides (MCTs).

#### 4.2.2. Preparation of LBG-RF Emulsion

The concentration of 10% Medium-Chain Triglycerides (MCT) was determined based on a balance of nutritional, rheological, and stability requirements for dysphagia management. Nutritionally, lipids are added to increase the caloric density of texture-modified foods. MCTs were specifically chosen because they are absorbed and metabolized more efficiently than long-chain fatty acids, and a 10% inclusion rate significantly boosts energy intake for high-risk populations without significantly altering the flavor profile [17,18]. From a processing standpoint, preliminary experiments showed that the LBG-RF thickener matrix effectively stabilized 10% oil through the formation of a viscous aqueous network that prevents droplet coalescence [29,47,48,49]. Higher oil concentrations were found to negatively impact the flow properties, potentially compromising the targeted IDDSI Level 3 classification [23,31]. The notation for the samples prepared in this experiment is outlined in Table 6.

### 4.3. Simulated Salivary System

#### Simulated Salivary Fluid Buffer (SSF Buffer) Preparation

The Simulated Salivary Fluid (SSF) buffer was prepared according to the method described by Minekus et al. [50], with minor modifications. The buffer contained the following final salt concentrations: 15.1 mmol/L KCl, 3.7 mmol/L KH_2_PO_4_, 13.6 mmol/L NaHCO_3_, 0.15 mmol/L MgCl_2_·6H_2_O, 0.06 mmol/L (NH_4_)_2_CO_3_, and 50 μL of 0.3 M CaCl_2_·2H_2_O stock solution per liter. The pH of the SSF buffer was adjusted to 7 with 1 M NaOH and 6 M HCl, then stored at −20 °C until use.

Following the protocol of Minekus et al. [50], the Simulated Salivary Fluid (α-SSF) system was prepared by adding α-amylase to the SSF buffer. The enzyme was added to achieve a final activity of 75 U/mL, and the mixture was then designated α-SSF. All solutions and samples were preheated to 37 °C in a water bath (Model B206). Next, either the SSF Buffer (without α-amylase) or the α-SSF was added to the sample at a 1:1 (*w*:*w*) ratio. This ratio follows the INFOGEST static in vitro digestion protocol, which standardized the oral phase to ensure reproducibility and to mimic the physiological transition of food into a bolus. The 1:1 dilution is designed to represent the typical volume of saliva incorporated into a bolus for standardized testing, facilitating accurate assessment of both the dilutive effect of salivary salts and the hydrolytic activity of α-amylase on the food matrix [50].

### 4.4. Viscosity Measurement

Viscosity measurements were carried out using a modified method based on Bolivar-Prados et al. [51]. A rotational viscometer with small, temperature-controlled adapters (APM) was used. Experimental conditions included a temperature of 37 °C and shear rates of 50 and 10 s^−1^. The apparent viscosity was recorded after 60 s. TL series cone-type rotors were employed: Spindle TL 6 for liquids with apparent viscosity between 0 and 200 mPa·s at 50 s^−1^, and Spindle TL 9 for liquids between 200 and 2000 mPa·s at 50 s^−1^. Torque was measured from the electronically recorded deflection angle, and the apparent viscosity (mPa·s) and shear stress (Pa) were calculated using the connected control unit.

### 4.5. Flow Properties Analysis

The flow behavior of selected thickened samples (L1R0.5 and calcium-modified thickened samples) was evaluated using a modified method from Salehi et al. [52]. Before adding α-SSF, samples were tested at fixed shear rates of 10, 20, 30, 40, and 50 s^−1^. After adding either SSF buffer or α-SSF, samples were analyzed at shear rates of 10, 30, 50, 70, and 85 s^−1^. Shear stress σ and shear rate $\dot{\gamma }$ data were recorded and fitted to three standard flow models to assess the fluid’s flow properties.

(1) $$\mathrm{Power}\;\mathrm{Law}\;\mathrm{Model}:\;\sigma =K\dot{\gamma^{n}}$$

(2) $$\mathrm{Herschel}–\mathrm{Bulkley}\;\mathrm{Model}:\;\sigma_{0}+K\dot{\gamma^{n}}$$

(3) $$\mathrm{Casson}\;\mathrm{Model}:\;\sigma^{0.5}={\sigma_{0}}^{0.5}+K_{c}{\dot{\gamma }}^{0.5}$$

where *σ* = shear stress (Pa), *σ*_0_ = yield stress (Pa), $\dot{\gamma }$ = shear rate (s^−1^), *K* = consistency coefficient (Pa.s), *K_c_* = Casson model parameter (Pa·s), *n* = flow behavior index (dimensionless).

### 4.6. IDDSI Level Measurement

The International Dysphagia Diet Standardization Initiative (IDDSI) framework includes manual and qualitative tests [53]. To establish objective standards for texture assessment, these empirical tests are combined with instrument-based, quantitative measurements, as explained below.

#### 4.6.1. Empirical (Syringe/Utensil) Analysis

Simple, non-instrumental tests were used to determine the IDDSI level of the food or drink samples [53]. For the syringe flow test, a 10 mL syringe with a measured length of 61.5 mm from the zero line to the 10 mL mark was used, with the plunger removed. The syringe tip was blocked with a finger, and the sample was filled to the 10 mL mark. The finger was then removed, and a 10-s timer was started. At the 10-s mark, the syringe tip was blocked again, and the remaining liquid volume (mL) was recorded. For the fork drip test: A four-tine fork with 4 mm spacing between the tines was immersed in the test liquid sample, withdrawn, and examined for flow and residue patterns. For the spoon tilt test, the residue on the spoon was observed as the spoon was tilted.

#### 4.6.2. Instrument-Assisted IDDSI (A-IDDSI/F and A-IDDSI/SC) Analysis

For quantitative measurements, a texture analyzer with a 5 kg load cell was used, along with an accessory fixture designed for mounting on the texture analyzer. This setup enabled controlled application of force and speed, as well as precise management of contact time and the positions of the fork and spoon relative to the food sample [54]. For the fork drip test (A-IDDSI/F) and spoon tilt test (A-IDDSI/SC), the respective fork or spoon attachment was installed and calibrated. The liquid sample was filled into the sample container up to the 200 mL mark and placed on the fixed base for measurement. In the fork drip test (A-IDDSI/F/F), the central probe holding the fork was set to contact the upper crossbar of the fixed base, thereby aligning the fork. It was then lowered to a consistent depth below the liquid surface, raised above the sample container, suspended for 5 s, and finally returned to the initial position. For the spoon tilt test (A-IDDSI/SC), the central probe holding the spoon was programmed to touch the upper crossbar of the fixed base, thereby leveling the spoon. The spoon was then moved to a consistent depth below the liquid surface, raised above the liquid surface, moved over the sample container, and paused for 5 s to weigh the adhered product. Finally, it was returned to the vertical position to discharge the product into the container.

### 4.7. Texture Profile Analysis (TPA)

To analyze the sample textures, the researchers used a method adapted from Yang et al. [55], ensuring the preparation met the standards set by Japan’s Ministry of Health, Labor and Welfare [56]. Each sample was carefully molded using molds with a 40 mm diameter and 20 mm height. Before testing, the molds were placed in a temperature-controlled copper fixture, allowing the samples to reach a consistent temperature of 37 °C—mimicking conditions relevant to food consumption. For measurement, a texture analyzer with a 5 kg load cell and a 20 mm cylindrical probe was used. The process began by filling the mold with the sample to a height of 5 mm. The probe was then positioned exactly 5 mm above the sample surface. Once set, the probe descended at a steady rate of 10 mm/s until it was 5 mm from the bottom of the mold. After reaching this point, the probe was gradually lifted back to its starting position, completing two cycles of compression. This systematic method ensured consistent and reliable texture measurements across all samples. The experiment was performed with five replicates. Four parameters—Hardness, Adhesiveness, Cohesiveness, and Gumminess—were calculated using Exponent Lite software (version 6). The samples were classified according to the standards for permitted food textures for dysphagia patients in Japan.

### 4.8. Microstructural Analysis

To stop α-amylase enzymatic activity, samples that had undergone the simulated salivary fluid (α-SSF) treatment were immediately placed in crushed ice for 10 min. Microstructural analysis of the emulsion was performed by placing a single drop of the emulsion between a microscope slide and a coverslip, as described by Lin [31]. The microstructures were observed using an optical microscope (Leica DM500, Leica Microsystems, Wetzlar, Germany) equipped with a 10× eyepiece and a 40× objective lens. The microscope was fitted with a 0–10 scale measuring reticle for size estimation.

### 4.9. Emulsion Stability

The emulsion stability test was performed using a modified method by Liu et al. [57]. Each emulsion sample was placed in a 15 mL centrifuge tube and stored at 4 °C. The separation height Hs and the total height of the emulsion (*H_t_*) were recorded after storage periods of 1, 7, 14, 21, and 28 days. The stability index (*SI*) was calculated using the following equation:

(4) $$SI\;\left(\%\right)=\frac{H_{s}}{H_{t}}\times 100$$

### 4.10. Statistical Analysis

The parameters for the fluid model regression equations (Power Law, Herschel–Bulkley, and Casson) were determined using SigmaPlot 14.0. All experimental data were analyzed with the Statistical Package for the Social Sciences (SPSS version 19.0). The statistical methods included one-way analysis of variance (ANOVA) to identify significant differences among the experimental groups. Duncan’s multiple-range test was then used for post hoc comparisons of means to pinpoint specific significant differences. Additionally, an independent samples *t*-test was performed to compare the two groups. A *p*-value of less than 0.05 was considered statistically significant unless otherwise noted.

## Figures and Tables

**Table 1:** Trends in concentration (*w*/*w*%) and apparent viscosity (mPa·s) of L1R0, L1R4, and L1R8 food emulsions.

	Model	Regression Equation	R^2^
L1R0.5	Power law	y = 174.330·x^2.915^	0.958
	Linear	y = 1920.178·x − 2486.283	0.985
L1R4	Power law	y = 34.633·x^2.497^	0.963
	Linear	y = 740.101·x − 1834.197	0.974
L1R8	Power law	y = 22.697·x^2.330^	0.955
	Linear	y = 515.285·x − 1605.783	0.974

y: apparent viscosity (mPa·s); x: concentration (*w*/*w*%). Formula names are shown in Section 4.2.2.

**Table 2:** Apparent viscosity of food emulsions at 50 s^−1^ and 10 s^−1^.

**Honey-like Emulsion**			
**Sample**	$\eta_{50}$ **(mPa·s)**	$\eta_{10}$ **(mPa·s)**	***t*** **-Test**
HL1R0.5	1046.27 ± 15.542 ^e^	2191.57 ± 52.871 ^e^	**
HL1R4	1040.93 ± 10.653 ^e^	2247.70 ± 3.110 ^e^	**
HL1R8	1027.90 ± 11.616 ^ef^	2321.37 ± 3.754 ^d^	**
**Pudding-like Emulsion**			
**Sample**	$\eta_{50}$ **(mPa·s)**	$\eta_{10}$ **(mPa·s)**	***t*** **-Test**
PL1R0.5	1811.03 ± 12.220 ^a^	4349.73 ± 61.617 ^a^	**
PL1R4	1761.43 ± 7.613 ^b^	4017.20 ± 63.288 ^b^	**
PL1R8	1757.97 ± 13.655 ^b^	3974.97 ± 24.241 ^b^	**
**Calcium-Modified Emulsion**			
**Sample**	$\eta_{50}$ **(mPa·s)**	$\eta_{10}$ **(mPa·s)**	***t*** **-Test**
CC-LR	1246.37 ± 10.017 ^c^	2649.13 ± 16.652 ^c^	**
LC-LR	1018.20 ± 5.841 ^f^	2085.60 ± 18.168 ^f^	**
GC-LR	1191.13 ± 16.374 ^d^	2615.23 ± 41.704 ^c^	**

*η*_50_ is the apparent viscosity measured at 50 shear rate (s^−1^)$;\;\eta_{10}$ is the apparent viscosity measured at a 10-shear rate (s^−1^). Formula names are shown in Section 4.2.2; the small letters in the same shear rate are significantly different (*p* < 0.05); ** indicates extremely significant difference (*p* < 0.01) between $\eta_{50}$ and $\eta_{10};$ values are mean ± standard deviation (n = 3).

**Table 3:** Power Law model and Casson model fit resolved for the food emulsions formulas.

Formula	Treatment	Power Law Model			Casson Model	
		*K* (Pa·s*^n^*)	*n*	R^2^	*σ*_0_ (Pa)	R^2^
HL1R0.5	Initial	7.66 ± 0.207	0.517 ± 0.0035	0.982	8.29 ± 0.238	0.970
	+SSF Buffer	0.45 ± 0.320	0.686 ± 0.2499	0.984	−0.71 ± 0.773	0.969
	+α-SSF	0.03 ± 0.000	1.147 ± 0.0000	0.999	0.65 ± 0.000	0.936
HL1R4	Initial	7.90 ± 0.259	0.489 ± 0.0084	0.994	9.15 ± 0.442	0.985
HL1R8	Initial	8.08 ± 0.576	0.485 ± 0.0150	0.991	9.45 ± 0.819	0.982
PL1R0.5	Initial	17.94 ± 0.327	0.434 ± 0.0025	0.988	21.30 ± 0.421	0.972
	+SSF Buffer	0.56 ± 0.308	0.701 ± 0.0916	0.989	−0.63 ± 0.893	0.969
	+α-SSF	0.74 ± 0.407	0.642 ± 0.1487	0.978	−1.65 ± 1.507	0.936
PL1R4	Initial	15.09 ± 0.052	0.455 ± 0.0280	0.997	18.06 ± 0.147	0.987
PL1R8	Initial	14.99 ± 0.292	0.458 ± 0.0036	0.999	18.13 ± 0.416	0.992
CC-LR	Initial	8.55 ± 0.276	0.515 ± 0.0059	0.991	9.47 ± 0.317	0.980
	+SSF Buffer	0.66 ± 0.028	0.561 ± 0.0161	0.966	−1.43 ± 0.235	0.969
	+α-SSF	0.04 ± 0.029	1.089 ± 0.0991	0.946	0.50 ± 0.270	0.936
LC-LR	Initial	6.60 ± 0.275	0.534 ± 0.0086	0.991	7.04 ± 0.442	0.980
	+SSF Buffer	0.03 ± 0.000	1.147 ± 0.0000	0.999	0.65 ± 0.000	0.969
	+α-SSF	0.23 ± 0.197	0.962 ± 0.6796	0.921	0.19 ± 0.820	0.936
GC-LR	Initial	9.93 ± 0.452	0.478 ± 0.0116	0.989	11.34 ± 0.695	0.975
	+SSF Buffer	0.11 ± 0.000	0.929 ± 0.0000	0.991	0.12 ± 0.000	0.969
	+α-SSF	0.05 ± 0.025	1.047 ± 0.0889	0.948	0.41 ± 0.235	0.936

Values are expressed as mean ± standard deviation. Formulas are defined in Section 4.2.2.

**Table 4:** A/IDDSI-F test and A/IDDSI-SC results.

Test	Sample	Initial Weight (g)	Weight at 5 s (g)
Fork drip	HL1R0.5	7.344 ± 0.3187 ^d^	1.598 ± 0.3050 ^ab^
	HL1R4	6.621 ± 0.5344 ^e^	0.875 ± 0.4803 ^d^
	HL1R8	7.167 ± 0.2209 ^d^	1.090 ± 0.1977 ^cd^
	PL1R0.5	9.871 ± 0.1569 ^b^	1.827 ± 0.1250 ^a^
	PL1R4	10.303 ± 0.1919 ^a^	1.327 ± 0.1758 ^bc^
	PL1R8	9.585 ± 0.1186 ^b^	1.376 ± 0.1691 ^bc^
	CC-LR	8.376 ± 0.0798 ^c^	1.208 ± 0.1550 ^c^
	LC-LR	8.083 ± 0.1685 ^c^	1.264 ± 0.1910 ^c^
	GC-LR	8.238 ± 0.0563 ^c^	1.116 ± 0.0859 ^cd^
Spoon tilt	HL1R0.5	7.344 ± 0.3187 ^d^	1.598 ± 0.3050 ^ab^
	HL1R4	6.621 ± 0.5344 ^e^	0.875 ± 0.4803 ^d^
	HL1R8	7.167 ± 0.2209 ^d^	1.090 ± 0.1977 ^cd^
	PL1R0.5	9.871 ± 0.1569 ^b^	1.827 ± 0.1250 ^a^
	PL1R4	10.303 ± 0.1919 ^a^	1.327 ± 0.1758 ^bc^
	PL1R8	9.585 ± 0.1186 ^b^	1.376 ± 0.1691 ^bc^
	CC-LR	8.376 ± 0.0798 ^c^	1.208 ± 0.1550 ^c^
	LC-LR	8.083 ± 0.1685 ^c^	1.264 ± 0.1910 ^c^
	GC-LR	8.238 ± 0.0563 ^c^	1.116 ± 0.0859 ^cd^

The superscript letters in the same column are significantly different (*p* < 0.05); values are mean ± standard deviation (n = 5); formula names are shown in Section 4.2.2.

**Table 5:** Texture characteristics analysis results of food emulsions.

Sample	Hardness	Adhesiveness	Cohesiveness	Gumminess	Level
HL1R0.5	400.0 ± 2.72 ^c^	39.0 ± 1.20 ^a^	1.0 ± 0.01 ^a^	403.5 ± 5.38 ^c^	III
HL1R4	217.3 ± 4.12 ^f^	32.9 ± 0.73 ^b^	0.96 ± 0.01 ^cd^	207.9 ± 1.76 ^g^	-
HL1R8	185.4 ± 1.66 ^g^	26.7 ± 2.29	0.911 ± 0.07 ^e^	168.9 ±10.00 ^h^	-
PL1R0.5	420.8 ± 1.36 ^b^	39.7 ± 1.26 ^a^	1.0 ± 0.01 ^ab^	422.5 ± 6.53 ^b^	III
PL1R4	464.1 ± 3.23 ^a^	41.0 ± 1.25 ^a^	0.99 ± 0.01 ^ab^	462.6 ± 6.44 ^a^	III
PL1R8	418.7 ± 4.16 ^b^	32.0 ± 2.14 ^b^	0.93 ± 0.027 ^de^	390.0 ± 14.28 ^d^	III
CC-LR	279.1 ± 4.95 ^e^	26.8 ± 2.39 ^c^	0.90 ± 0.00 ^abc^	276.4 ± 3.70 ^f^	-
LC-LR	278.2 ± 7.50 ^e^	22.7 ± 0.86 ^d^	0.97 ± 0.007 ^bc^	269.9 ± 8.76 ^f^	-
GC-LR	305.4 ± 4.49 ^d^	22.6 ± 1.91 ^d^	0.97 ± 0.005 ^abc^	297.2 ± 4.60 ^e^	III

Unit of hardness (N/m^2^), Adhesiveness(J/m^3^). Sample abbreviations are shown in Section 4.2.2. The superscript letters in the same column are significantly different (*p* < 0.05). Values are mean ± standard deviation (n = 5).

**Table 6:** Sample abbreviation followed by the specific formulation.

**Honey-like Emulsion**					
**Sample Abbreviation**	**Hydrocolloid**				**Oil**
	**L**		**R**		**MCT**
HL1R0.5	1.22%		0.61%		10%
HL1R4	0.78%		3.12%		
HL1R8	0.57%		4.56%		
**Pudding-like Emulsion**					
	**Hydrocolloid**				**Oil**
	**L**		**R**		**MCT**
PL1R0.5	1.50%		0.75%		10%
PL1R4	0.98%		3.92%		
PL1R8	0.73%		5.84%		
**Calcium-Modified Emulsion**					
	**Ca^+2^ Source**	**Hydrocolloid**			**Oil**
		**L**		**R**	**MCT**
CC-LR	4.5 g CC	1.22%		0.61%	10%
LC-LR	4.5 g LC				
GC-LR	4.5 g GC				

H: Honey-like; P: pudding-like; L: locust bean gum; R: pre-gelatinized rice flour; CC: carbonate-orient calcium source; LC: lactates-oriented calcium source; GC: gluconate-orient calcium source; MCT: medium-chain triglycerides.

## Data Availability

Data available upon request from the editorial team.

## Appendix B

**Table A1:** Herschel-Bulkley model fit results for food emulsions.

Herschel–Bulkley Model				
**Native Emulsion**				
	$\sigma_{0}$ **(Pa)**	***K*** **(Pa·s*^n^*)**	***n***	**R^2^**
HL1R0.5	−157.75 ± 87.592	143.22 ± 83.704	0.117 ± 0.0396	0.992
HL1R4	−31.74 ± 15.490	30.37 ± 12.290	0.273 ± 0.0531	0.997
HL1R8	−22.52 ± 1.412	23.16 ± 0.420	0.303 ± 0.0009	0.993
PL1R0.5	−429.86 ± 140.206	413.46 ± 137.292	0.066 ± 0.0194	0.997
PL1R4	−46.81 ± 14.341	48.67 ± 11.210	0.266 ± 0.0346	0.999
PL1R8	−13.57 ± 7.195	23.63 ± 4.692	0.380 ± 0.0368	0.999
CC-LR	−68.95 ± 25.405	60.55 ± 22.800	0.207 ± 0.0422	0.996
LC-LR	−70.20 ± 30.993	60.56 ± 27.503	0.191 ± 0.0462	0.998
GC-LR	−231.21 ± 19.808	216.32 ± 20.502	0.079 ± 0.0068	0.998
**Emulsion with SSF Buffer**				
HL1R0.5	−0.71 ± 0.773	0.45 ± 0.320	0.686 ± 0.2499	0.984
PL1R0.5	−0.63 ± 0.893	0.56 ± 0.308	0.701 ± 0.0916	0.989
CC-LR	−1.43 ± 0.235	0.66 ± 0.028	0.561 ± 0.0161	0.966
LC-LR	0.65 ± 0.000	0.03 ± 0.000	1.147 ± 0.0000	0.999
GC-LR	0.12 ± 0.000	0.11 ± 0.000	0.929 ± 0.0000	0.991
**Emulsion with α-SSF Buffer**				
HL1R0.5	0.65 ± 0.000	0.03 ± 0.000	1.147 ± 0.0000	0.999
PL1R0.5	−1.65 ± 1.507	0.74 ± 0.407	0.642 ± 0.1487	0.978
CC-LR	0.50 ± 0.270	0.04 ± 0.029	1.089 ± 0.0991	0.946
LC-LR	0.19 ± 0.820	0.23 ± 0.197	0.962 ± 0.6796	0.921
GC-LR	0.41 ± 0.235	0.05 ± 0.025	1.047 ± 0.0889	0.948

Values are mean ± standard deviation (n = 3). Formulas are shown in Table 6.
